# Dynamic Gelatin Hydrogels Crosslinked by Dithiolane‐Norbornene Click Chemistry

**DOI:** 10.1002/mabi.202500400

**Published:** 2026-02-03

**Authors:** Favour O. Afolabi, Lydia Yang He, Chien‐Chi Lin

**Affiliations:** ^1^ Weldon School of Biomedical Engineering Purdue University West Lafayette Indiana USA

**Keywords:** 3D/4D cell culture, dithiolanes, dynamic hydrogels, gelatin‐norbornene, induced pluripotent stem cells, lipoic acid

## Abstract

Hydrogels prepared from gelatin are ideal for mimicking the extracellular matrix (ECM) owing to their inherent cell‐adhesive and protease‐labile peptide sequences. While gelatin is highly water‐soluble, it does not form the triple‐helical structure. As a result, physically crosslinked gelatin‐based hydrogels are only stable at low temperatures, precluding their use in 3D cell culture. Gelatin‐methacryloyl (GelMA) and gelatin‐norbornene (GelNB) have been developed to enable the stable crosslinking of gelatin‐based hydrogels via chain‐growth or step‐growth photopolymerization. However, most gelatin‐based hydrogels lack dynamically tunable properties unless macromers with dynamically crosslinkable motifs are used. Here, we integrate GelNB with dithiolane‐containing crosslinker poly(ethylene glycol)‐tetra‐lipoic acid (PEG4LA)‐for modular photo‐crosslinking of GelNB into hydrogels under cytocompatible light exposure (365 nm, 5 mW/cm^2^) with a low photoinitiator concentration (1 mm LAP). Even under these mild reaction conditions, the stiffness of GelNB/PEG4LA hydrogels could be dynamically tuned by inducing dithiolane ring‐opening via secondary light exposure, thereby creating dynamic and cytocompatible hydrogels suitable for in situ encapsulation, culture, and differentiation of human induced pluripotent stem cells (hiPSCs).

## Graphical abstract

Gelatin‐norbornene (GelNB) and poly(ethylene glycol)‐tetra‐lipoic acid (PEG4LA) are modularly photo‐crosslinked into gelatin‐based hydrogels with dynamically tunable mechanics for expansion and differentiation of induced pluripotent stem cells (iPSC).

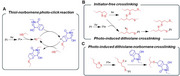

## 1 Introduction

Human induced pluripotent stem cells (hiPSCs) are valuable in disease modeling and tissue regeneration as they can differentiate into all three germ layers [1, 2]. Most hiPSC differentiation protocols, however, focus on optimizing soluble cues and extracellular matrices (ECM) coating [3, 4], while neglecting the effect of time‐dependent changes in matrix mechanics. In 3D space, ECM may become stiffer due to deposition of collagen, hyaluronic acid (HA), and other ECM molecules [5]. On the other hand, increased expression of matrix metalloproteinases (MMPs) leads to matrix degradation and softening. Both matrix stiffening and softening can trigger intracellular signaling events that alter cell fate processes [6]. Traditional cell culture substrates, such as tissue culture plastics (TCP), are easy to use but lack physiological relevance, as their surfaces exhibit ultrahigh substrate stiffness (> GPa). Moreover, TCP does not recapitulate the dynamic 3D microenvironment. Derived from the basement membrane of murine sarcoma, Matrigel is commonly used as a surface coating or 3D matrix for the culture and differentiation of hiPSCs. However, Matrigel has no translational potential owing to its tumor origin. Additionally, Matrigel is not a robust matrix due to its high batch variability, undefined biochemical composition [7], and limited tunability [8]. These drawbacks have driven growing interest in chemically crosslinked biomaterial scaffolds, which offer greater control over matrix mechanics and improved reproducibility.

Hydrogel matrices can be crosslinked to achieve the desired biophysical and biochemical properties, emulating the properties of native ECM [9, 10]. For example, chemically crosslinked gelatin hydrogels have been extensively used in tissue engineering and regenerative medicine applications [11, 12]. Derived from denatured collagen, gelatin contains various integrin binding motifs (e.g., arginine‐glycine‐aspartic acid or RGD) [13] and protease‐labile sequences [14]. However, gelatin has an Upper Critical Solution Temperature (UCST) above room temperature and requires modification to render it stably crosslinked at body temperature [15]. Gelatin‐based hydrogels can be crosslinked by chain‐growth photopolymerization of gelatin‐methacryloyl (GelMA) [16], which can be initiated by Irgacure 2959 or lithium acylphosphinate salt (LAP). While chain‐growth polymerization is easy to perform, it generates a high concentration of radical species during polymer chain propagation, which are detrimental to sensitive cell types. Alternatively, step‐growth polymerized hydrogels, such as gelatin‐norbornene (GelNB) gels crosslinked by orthogonal thiol‐norbornene click chemistry, enable efficient crosslinking with lower photoinitiator concentrations, rapid reaction kinetics, and high cytocompatibility [17]. For example, Arkenberg et al. demonstrated in situ encapsulation and trilineage (mesoderm, endoderm, and neuroectoderm) differentiation of hiPSC in GelNB thiol‐norbornene hydrogels [18]. Despite their respective advantages, hydrogels crosslinked by conventional GelMA and GelNB contain non‐dynamic polymethacrylate [16] and thioether bond [17], respectively, limiting their use in the dynamic hydrogel space. To address this limitation, our group and others have reported gelatin‐based hydrogels with dynamically tunable properties, including gels that can be stiffened by orthogonal click reactions [19], enzymatic reactions [20], light‐based reactions [21, 22, 23], and dynamic covalent chemistries (DCC) [24]. These platforms have been used to study tumor progression, stem cell fate, and fibroblast behavior. For example, Liu et al. synthesized GelNB‐hydroxyphenylacetic acid (GelNB‐HPA) hydrogels and used tyrosinase to temporally stiffen the hydrogels to study the effect of matrix stiffness on activation of pancreatic stellate cells [20]. Nguyen et al. utilized GelNB‐HPA alongside GelNB‐boronic acid (GelNB‐BA) to induce viscoelastic stiffening using tyrosinase to investigate the role of dynamic matrix properties on pancreatic cancer cell behavior [24]. Chang et al. synthesized GelNB‐carbohydrazide (GelNB‐CH) hydrogels amenable to thiol‐norbornene crosslinking and hydrazone‐induced dynamic stiffening, allowing for concurrent HA accumulation and matrix stiffening [19]. Nonetheless, these approaches required dual modification of gelatin to enable orthogonal control of primary gel crosslinking and secondary stiffening.

Dynamic covalent chemistry (DCC) offers an alternative for dynamically controlling gel properties. With DCC, chemical bonds formed after primary crosslinking remain adaptable for secondary reactions, such as network reconfiguration or ligand immobilization. Dithiolane‐mediated ring‐opening polymerization (ROP) is an emerging DCC for fabricating dynamic hydrogels [25, 26, 27], For example, Scheutz et al. synthesized linear PEG‐phenyl‐1,2‐dithiolane (PEG‐PhDL) for initiator‐free photocrosslinking [26]. However, gelation was slow and inefficient, with soft gels (< 1 kPa) formed after 10 min of light irradiation (at an unknown intensity) at room temperature. Moreover, the dynamic features imparted by dithiolane's reversible thiol‐disulfide exchange were not explored. Nelson et al. incorporated a norbornene‐modified polymer to accelerate the crosslinking of a dithiolane‐based hydrogel [27]. With additional LAP and dithiolane moieties, these hydrogels could be dynamically stiffened upon secondary light exposure. Khine et al. synthesized linear synthetic polymers containing both lipoic acid and boronic acid [25], enabling initiator‐free photocrosslinking (365 nm, 20 mW/cm^2^, 5 min) to form viscoelastic hydrogels. Nonetheless, pure dithiolane‐based hydrogelation typically requires high light intensity and elevated temperatures [25, 28], causing potential cytotoxicity to the encapsulated cells. More importantly, these synthetic hydrogels were inert to cells, necessitating the inclusion of cell‐adhesive peptides and protease‐labile motifs to facilitate cell‐matrix interactions. In a recent work, Asim et al. conjugated lipoic acid to gelatin, yielding gelatin‐lipoic acid (Gel‐LA) hydrogel that supported 3D culture of fibroblasts and tumoroids [29]. However, direct LA modification on gelatin significantly reduced gelatin solubility, and it is unclear if the stiffness of Gel‐LA hydrogels can be dynamically tuned. Furthermore, none of the prior dithiolane‐based hydrogels were used for the culture and differentiation of hiPSCs [27, 30].

In this work, we report the development and characterization of a gelatin‐based dithiolane‐norbornene hydrogel system with on‐demand tunable mechanics for culture and definitive endoderm differentiation of hiPSCs. By copolymerizing GelNB with tetrafunctional PEG‐Lipoic Acid (PEG4LA) under mild reaction conditions, 3T3 fibroblasts and hiPSCs were safely photo‐encapsulated in these gels. Furthermore, cell‐laden GelNB/PEG4LA hydrogels were dynamically stiffened via light‐induced disulfide exchange, enabling investigation of how dynamic mechanical changes affect stem cell behavior in vitro.

## 2 Materials and Methods

### 2.1 Materials

Type A gelatin (238‐282 bloom) was purchased from VWR (TS61199‐5000). 4‐arm PEG‐Amine (PEG4NH_2_, 20 kDa) and 8‐arm PEG‐Hydroxyl (PEG8OH, 20 kDa) were purchased from JenKem Technology USA. 4‐arm PEG‐Thiol (PEG4SH, 10 kDa) was acquired from Laysan Bio. Lithium phenyl‐2,4,6‐trimethylbenzoylphosphinate (LAP), methanol, and dimethyl sulfoxide (DMSO) were purchased from Sigma–Aldrich. Carbic anhydride (A134511000), Dichloromethane (DCM, 99.8%), and N‐(3‐Dimethylaminopropyl)‐N’‐ethylcarbodiimide (EDC) hydrochloride were purchased from Acros Organics. O‐(7‐Azabenzotriazole‐1‐yl)‐N, N, N, N’‐tetramethyluronium hexafluorophosphate (HATU) was purchased from Chem‐Implex International. Methylmorpholine (AC127151000) and triethylamine (O48851) were purchased from Fischer Scientific. DL‐alpha‐Lipoic acid (>99%) was purchased from TCI (L00585G). HyClone Dulbecco's Modified Eagle Medium (DMEM, SH30243.01) and 100× antibiotic‐antimycotic solution (SV30079.01) were acquired from Cytiva. Dulbecco's phosphate‐buffered saline (DPBS) and fetal bovine serum (FBS, USDA Approved Origins) were purchased from Corning. 0.5% Trypsin‐EDTA was purchased from ThermoFisher (15400‐054). A definitive endoderm differentiation kit was purchased from STEMCELL Technologies. SOX17 (D1T8M, 1:200 dilution) Rabbit mAb was purchased from Cell Signaling Technology. Alexa Fluor 488 Donkey anti‐Rabbit IgG (H+L) (1:200 dilution) was purchased from Invitrogen (A21206). Rhodamine phalloidin (#PHDR1) was purchased from Cytoskeleton, Inc. DAPI (#AS‐83210) was purchased from AnaSpec. Rhodamine PEG‐Thiol (3.4 kDa) was purchased from NANOCS.

### 2.2 Macromer Synthesis and Characterization

#### 2.2.1 Gelatin‐Norbornene

GelNB was synthesized according to our published protocols with slight modifications [17]. Briefly, type‐A gelatin (1 g) was dissolved at 4% w/v in DPBS and functionalized with norbornene by reacting its primary amines with carbic anhydride (0.6 g) in the presence of triethylamine (2.86 mL) at a 1:1 molar ratio, a base catalyst, at room temperature overnight. The crude product was purified via dialysis (12–14 kDa MWCO) against deionized water, freeze‐dried (−50°C, 20 Pa), and stored at −20°C. Norbornene modification was verified by ^1^H‐NMR (400 MHz), and the degree of modification was quantified using a fluoraldehyde assay, which was approximately 90% on available amine groups (i.e., 5.4 mm of NB/wt%).

#### 2.2.2 Multi‐Armed Poly(ethylene glycol)‐Lipoic Acid (PEGLA)

PEG4NH_2_ (1 g, 200 µmol NH_2_) was dissolved in anhydrous DMF (6 mL), followed by the addition of HATU (304 mg, 800 µmol). Separately, lipoic acid (165 mg, 800 µmol) was dissolved in anhydrous DMF (4 mL) to prevent agglomeration and added to the PEG4NH_2_/HATU mixture. The solution was purged with nitrogen gas for 15 min, and methylmorpholine (162 mg, 1.6 mmol) was added dropwise under nitrogen, followed by an additional 10‐min purge. The reaction was conducted in the dark at room temperature for 48 h, after which it was terminated by air exposure. The crude product was purified by dialysis (3.5 kDa MWCO) against ACS‐grade DMF for 3 days, followed by deionized water for 2 days. The purified PEG4LA was freeze‐dried (−50°C, 20 Pa) and stored at −20°C. Modification of LA on PEG4NH_2_ was confirmed via ^1^H‐NMR (400 MHz).

### 2.3 Fabrication and Mechanical Characterization of GelNB Hydrogels

Hydrogel precursor solutions were cast between hydrophobic‐treated glass slides, forming gels ∼8 mm in diameter and 1 mm in thickness. The hydrogel dimensions were selected to fit the Anton Paar rheometer's parallel‐plate probe. The hydrogel precursor solutions were photoirradiated for 2 min (365 nm, 5 mW/cm^2^) and then incubated in DPBS for at least 1 h before rheological testing to achieve equilibrium swelling. Photopolymerization kinetics of non‐dynamic and dynamic hydrogel precursors were assessed using in situ photorheometry at a constant frequency (1Hz), strain (1%), and normal force (0N). Hydrogel stiffness was characterized with strain sweep rheometry at constant frequency (1Hz), 0.1%–5% strain, and normal force (0.25 N). Hydrogel viscoelasticity was characterized via stress‐relaxation tests using the same parameters as those used in strain‐sweep tests. Frequency sweep tests were conducted at constant strain (1%) with increasing angular frequency (0.1–100 rad/s) to confirm stiffness measurements within the linear viscoelastic range (LVR).

### 2.4 Dynamic Stiffening of GelNB/PEGLA Hydrogels

Dynamic stiffening of GelNB/PEG4LA hydrogels was performed using secondary light exposure and LAP incubation. Soft gels were incubated in buffer solutions containing no LAP, LAP‐only, or varied LAP (2.5, 5, 7.5 mm), or LAP (2.5 mm) plus PEG4LA (2.5, 5, 10% w/v) for 60 min at 37°C. This was followed by light exposure (365 nm, 5 mW/cm^2^). Real‐time stiffening was characterized using in situ photorheology, and cell‐laden gel stiffening was conducted using a bench UV lamp. To elucidate the effect of temperature on dynamic stiffening, groups of gels were irradiated at ambient (25°C) and elevated temperatures (37°C).

### 2.5 Enzymatic Degradation of GelNB/PEGLA Hydrogels

To assess the proteolytic degradation of GelNB/PEGLA hydrogels, preformed gels were incubated in 100 U/mL type I collagenase at 37°C, with mass loss measured every 30 min. The degradation profile was compared to GelNB/PEG4SH hydrogels.

### 2.6 Culture and Encapsulation of 3T3‐GFP Fibroblasts and Dynamic Stiffening of 3T3‐Laden Hydrogels

GFP‐transfected mouse embryonic fibroblasts (3T3‐GFP) were cultured in high‐glucose DMEM supplemented with 10% FBS and 1% P/S on tissue culture plates at 37°C, 5% CO_2_. Cells were passaged at ∼80%–90% confluency every 3–4 days. Cells (5 million/mL) were mixed with GelNB/PEG4LA hydrogel precursor, loaded into open‐cut syringes, and photo‐crosslinked (365 nm, 5 mW/cm^2^, 2 min). The cell‐laden gels were transferred to a 48‐well plate with 1 mL culture media and maintained for 7 days with media changes every other day.

For dynamic stiffening studies, cell‐laden gels were cultured for 2 days to allow cell acclimation to the matrix before stiffening. Gels were incubated in culture medium supplemented with 2.5 mm LAP for 1 h at 37°C, 5% CO_2_, then photoirradiated (365 nm, 5 mW/cm^2^). After stiffening, the cell‐laden gels were cultured for five more days, with media changes every other day. Cytoskeletal organization in non‐stiffened and stiffened gels was assessed using F‐actin staining. Gels were washed in PBS (3×, 10 minutes), fixed in paraformaldehyde (PFA) (2 h), and permeabilized in Triton X‐100 (2 h at room temperature or overnight at 4°C). Gels were incubated in rhodamine phalloidin for 2 h and DAPI for 1 h, followed by imaging using Andor's benchtop confocal microscope (BC‐43, Oxford Instruments).

### 2.7 Culture and Encapsulation of hiPSCs and Dynamic Stiffening of iPSC‐Laden Hydrogels

Cellartis iPSC‐12 (ChiPSC12), purchased from Takara, were cultured on tissue culture plates, precoated with vitronectin for at least 60 min at room temperature or overnight at 4°C. Cells were maintained in Essential 8 media (E8), purchased from ThermoFisher Scientific, following the manufacturer's directions. Cells were passaged at ∼80%–90% confluency every 4–5 days by dissociation with TrypLE (Gibco), incubation for 5 min (37°C, 5% CO_2)._ To enhance cell survival and prevent anoikis, freshly passaged cells were cultured in cell culture medium supplemented with 10 µm rock inhibitor (Y‐27632) for 24 h.

After cell dissociation and counting, ChiPSC12 were encapsulated in GelNB/PEG4LA hydrogels. Gently, the cells were mixed with the hydrogel precursor solution containing GelNB, PEG4LA, and LAP at a density of 5 million cells per mL. Cell‐laden gels were cultured in Essential 8 medium with 10 µm Y‐27632, with daily media changes. Viability was assessed using a live/dead staining kit (Life Technologies). iPSC‐laden gels were incubated in calcein AM and ethidium homodimer for 1 h at 37°C, followed by PBS washes (2×, 5 minutes).

For dynamic stiffening studies, iPSC encapsulation was performed as described above. Soft and stiffened hydrogels contained the same formulation (5% w/v GelNB, 5% w/v PEG4LA, and 1 mm LAP). Dynamic stiffening was initiated on day 2 of culture by adding 2.5 mm LAP to E8 media and incubating the cell‐laden gels for 60 minutes (37°C, 5% CO_2_), followed by photoirradiation. iPSC viability and pluripotency in stiffened gels were determined as described above and compared to non‐stiffened hydrogels.

### 2.8 Definitive Endoderm (DE) Differentiation of iPSC‐Laden Hydrogels

ChiPSC12 cells were encapsulated in GelNB/PEG4LA hydrogels and dynamically stiffened as outlined in Section 2.7. DE differentiation was initiated on day 4 of culture (i.e., two days after stiffening) using the STEMCELL Technologies definitive endoderm kit and continued for two days in both soft and stiffened gels. On day 6 of culture (i.e., two days of differentiation), cells were liberated from the gels via collagen digestion, followed by RNA extraction for quantitative real‐time PCR (qRT‐PCR) analysis of DE markers SOX17 and FOXA2. To increase RNA yield, iPSCs were liberated from at least three hydrogels using 100 U/mL Type I collagenase, followed by DPBS washes. RNA was isolated using the NucleoSpin RNA kit (MACHEREY‐NAGEL) and quantified using a DS‐11 FX+ spectrophotometer (DeNovix). cDNA synthesis was performed with the PrimeScript RT reagent kit, and pluripotency gene expression was analyzed using SYBR Premix Ex TaqII. Gene expression was normalized to GAPDH using the 2‐ΔΔCt method, comparing soft and stiffened gels to using 2D controls or soft gels as controls.

For immunostaining, cell‐laden hydrogels were washed with DPBS (3×, 10 minutes each), fixed in paraformaldehyde for 2 h, and blocked/permeabilized overnight at 4°C in a solution containing 1% BSA and 0.3% Triton X‐100. Samples were incubated overnight at 4°C with primary anti‐SOX17 (Rabbit) antibody, followed by three 30‐minute washes in the same blocking solution. The gels were then incubated with an Alexa Fluor 488‐conjugated anti‐rabbit secondary antibody overnight at 4°C, washed again (3×, 30 minutes), counterstained with DAPI for 1 h at room temperature, and imaged using Andor's benchtop confocal microscope (BC‐43, Oxford Instruments).

### 2.9 Statistics

All numerical data analyses and statistical analyses were performed using GraphPad Prism 10 software. Each experiment was repeated at least three times. Differences between groups were determined by one‐way or two‐way analysis of variance (ANOVA). One‐way ANOVA was performed to determine the statistical significance of the storage moduli of stiffened hydrogels in relation to non‐stiffened hydrogels. One‐way ANOVA was used to establish significance over the culture duration of 3T3 cells. Two‐way ANOVA was used to compare groups of data to establish significance. Two‐way ANOVA was used to establish significance in the area fraction of 3T3‐laden non‐stiffened and stiffened GelNB/PEG‐4LA. Datasets are presented as mean ± standard deviation (SD). The significance levels are indicated by asterisks: one asterisk (^*^) denotes *p* < 0.05, two asterisks (^**^) denote *p* < 0.01, three asterisks (^***^) denote *p* < 0.001, and four asterisks (^****^) denote *p* < 0.0001.

## 3 Results and Discussion

### 3.1 Synthesis and Crosslinking of Gelatin‐Based Thiol‐Ene (GelNB/PEG4SH) Hydrogels

**FIGURE 1:**
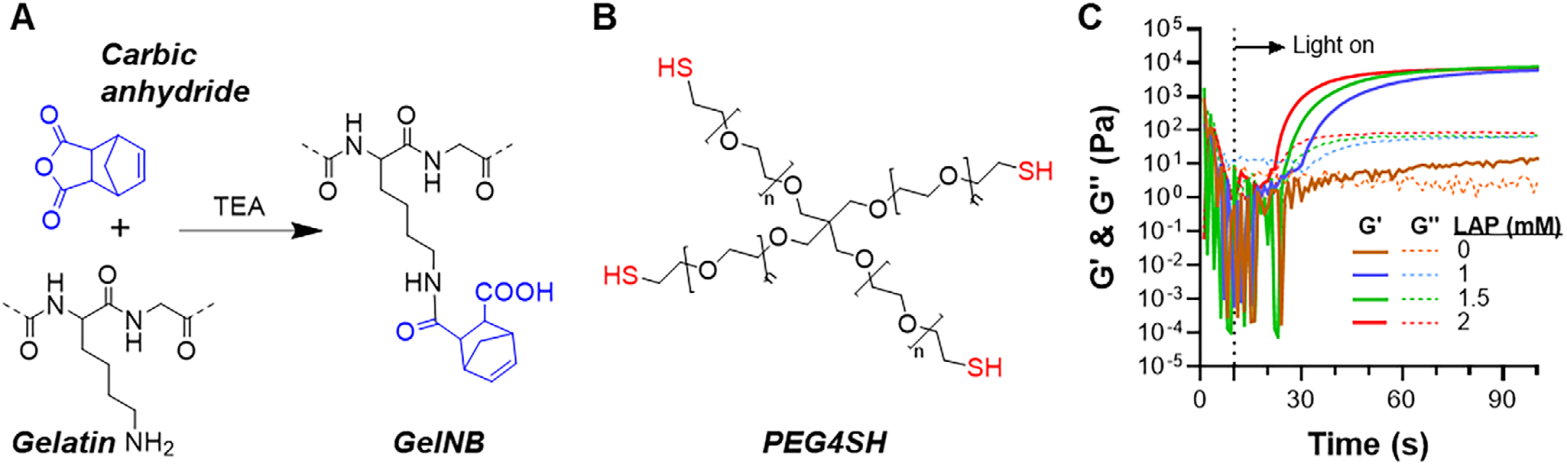
Synthesis and photopolymerization of gelatin‐based thiol‐norbornene hydrogels. (A) Modification of gelatin with carbic anhydride using TEA as the base catalyst to yield gelatin norbornene (GelNB). (B) Four‐arm PEG‐Thiol (PEG4SH) as the crosslinker. (C) In situ photo‐rheometry of GelNB/PEG4SH (both at 5% w/v) crosslinking with varying photoinitiator LAP concentrations. Longwave UV light (365 nm, 5 mW/cm^2^) was turned on 10 s after initiating the time‐sweep in situ photorheometry test.

**SCHEME 1:**
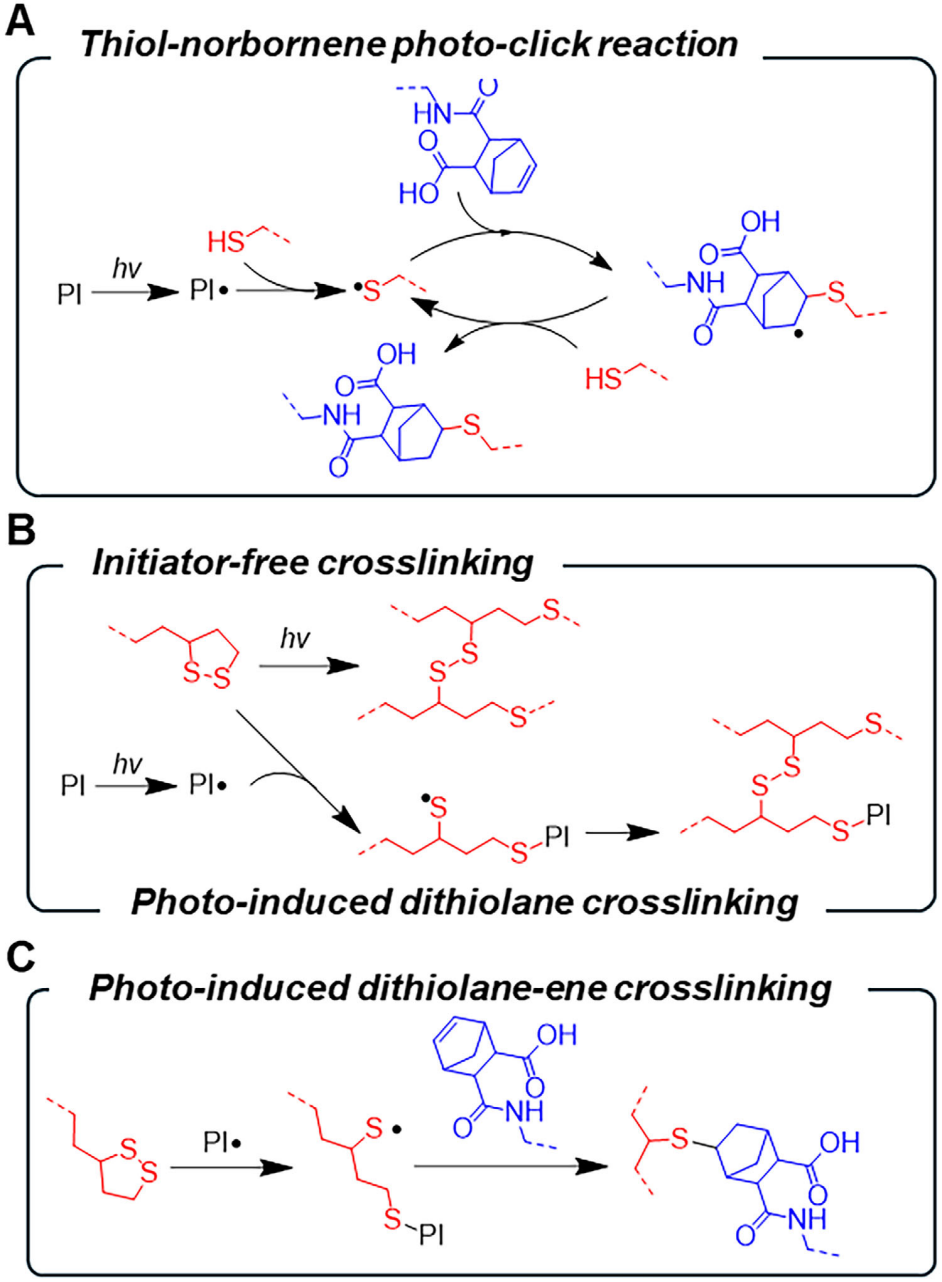
Crosslinking chemistry was used in this study. (A) Thiol‐norbornene photo‐click reaction. (B) Initiator‐free and photoinitiator‐induced dithiolane crosslinking. (C) Photoinitiator‐induced dithiolane‐norbornene crosslinking.

Our group previously demonstrated the synthesis of GelNB via reacting primary amine groups on pristine gelatin with carbic anhydride in a basic aqueous solution [17]. We further demonstrated enhanced control over norbornene substitution by using TEA as the base catalyst (Figure 1A). In this work, we synthesized GelNB with TEA as the base catalyst and obtained ∼ 90% of norbornene substitution (ca. ∼5.4 mm of NB/wt% Gelatin) (Figure S1). We first assessed the effectiveness of GelNB for hydrogel crosslinking using standard thiol‐norbornene photopolymerization with 4‐arm PEG‐thiol (PEG4SH; 10 kDa; 4 mm SH per wt%) as the crosslinker (Figure 1B) and photoinitiator LAP at various concentrations. In the conventional thiol‐norbornene photo‐click reaction (Scheme 1A), light irradiation breaks LAP (type 1 photoinitiator) into two radical species (PI•). One PI• abstracts a proton from a thiol group (─SH), creating a thiyl radical (─S•) that rapidly propagates across norbornene to form a stable thioether linkage and a carbon‐centered radical [17]. The latter terminates via abstracting a proton from another thiol, completing the step‐growth polymerization [17]. LAP is a highly water‐soluble and cytocompatible photoinitiator [31], and no noticeable gelation occurred without LAP. On the other hand, increasing LAP concentration (1–2 mm) accelerated gelation, as indicated by faster gel points (the time when storage modulus G′ surpasses loss modulus G″), from ∼20 to ∼10 s (Figure 1C). As all hydrogel precursors contained the same amount of GelNB and PEG4SH (5% w/v each), all gelation achieved similar final storage moduli of ∼8 kPa after ∼80 s of photoirradiation.

### 3.2 Crosslinking of Pure Dithiolane Hydrogels

**FIGURE 2:**
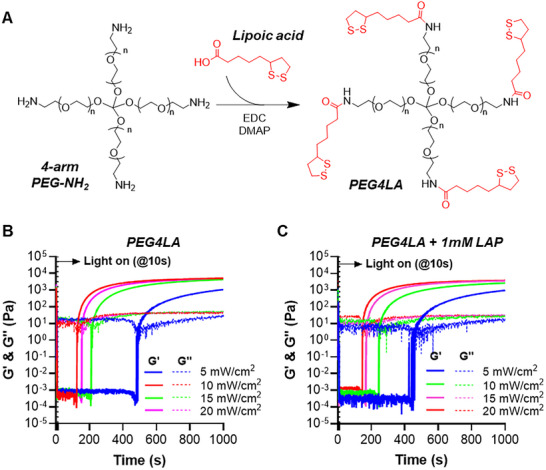
Synthesis and photopolymerization of pure dithiolane hydrogels. (A) Schematic of PEG4LA synthesis from reacting 4‐arm PEG‐amine with lipoic acid. (B, C) In situ photo‐rheometry of initiator‐free (B) and LAP (1 mm, ca. 0.029 wt%) mediated (C) photocrosslinking of 5% w/v PEG4LA. Light (365 nm) intensity was adjusted to 5, 10, 15, or 20 mW/cm^2^.

While thiol‐norbornene photocrosslinking between GelNB and PEG4SH was efficient, the thioether bonds formed between norbornene and thiol moieties were covalent and non‐dynamic (Scheme 1A). We hypothesize that replacing PEG4SH with a dithiolane‐containing PEG‐tetra‐lipoic acid (PEG4LA, Figure 2A; Figure S2A) will endow the GelNB hydrogels with dynamically tunable properties (Scheme 1B). However, pure dithiolane ROP often requires high polymer content (e.g., > 20% w/v), long light exposure time (e.g., > 5 min), or high light intensity (>10 mW/cm^2^), which are incompatible with in situ cell encapsulation [32]. Indeed, in the absence of LAP, 5% w/v PEG4LA precursors failed to form gel after 2 min of mild light exposure (365 nm, 5 mW/cm^2^, 2 min, Figure S2B). Dithiolane ROP can be accelerated by adding a photoinitiator and light irradiation, which generates reactive radicals to produce thiyl radicals from the dithiolane moiety. The thiyl radicals can then participate in thiol‐disulfide exchange (Scheme 1B) or thiol‐norbornene crosslinking (Scheme 1C). We first assessed the crosslinking of pure dithiolane hydrogels using just PEG4LA (5% w/v). Interestingly, adding 1 mm LAP did not lead to gelation under the desired mild light exposure (e.g., 365 nm at 5 mW/cm^2^, Figure S2B). As the red food dye added for visualizing the tube gelation test might adversely affect gelation, in situ photorheometry was conducted without food dye. Results showed that the gelation of PEG4LA did not occur until ∼490 s of 365 nm light exposure at 5 mW/cm^2^, either in the absence (Figure 2B) or presence of 1 mm LAP (Figure 2C). This result suggests that the crosslinking of pure dithiolane hydrogel was too slow for efficient cell encapsulation. This is consistent with previous work where a bifunctional dithiolane polymer could not reach gelation in the absence of LAP, and a tetrafunctional dithiolane polymer only reached slow gelation at relatively high light intensity (25 mW/cm^2^) and polymer concentration (> 20% w/v) [27]. While the crosslinking of pure PEG4LA was accelerated (gel points >130 s) at higher light intensity (10 to 20 mW/cm^2^, Figure 2B), adding 1 mm LAP did not further accelerate gelation (Figure 2C). Interestingly, at much higher LAP concentrations (i.e., 5 and 10 mm LAP), gelation of PEG4LA occurred rapidly, but the abundance of radical species caused reverse gelation (Figure S2D). This was likely due to the continued breakage of disulfide bonds and subsequent termination of the thiyl radicals by abundant LAP fragments [33].

### 3.3 Crosslinking of Gelatin‐Based Dithiolane‐Norbornene Hydrogels

**FIGURE 3:**
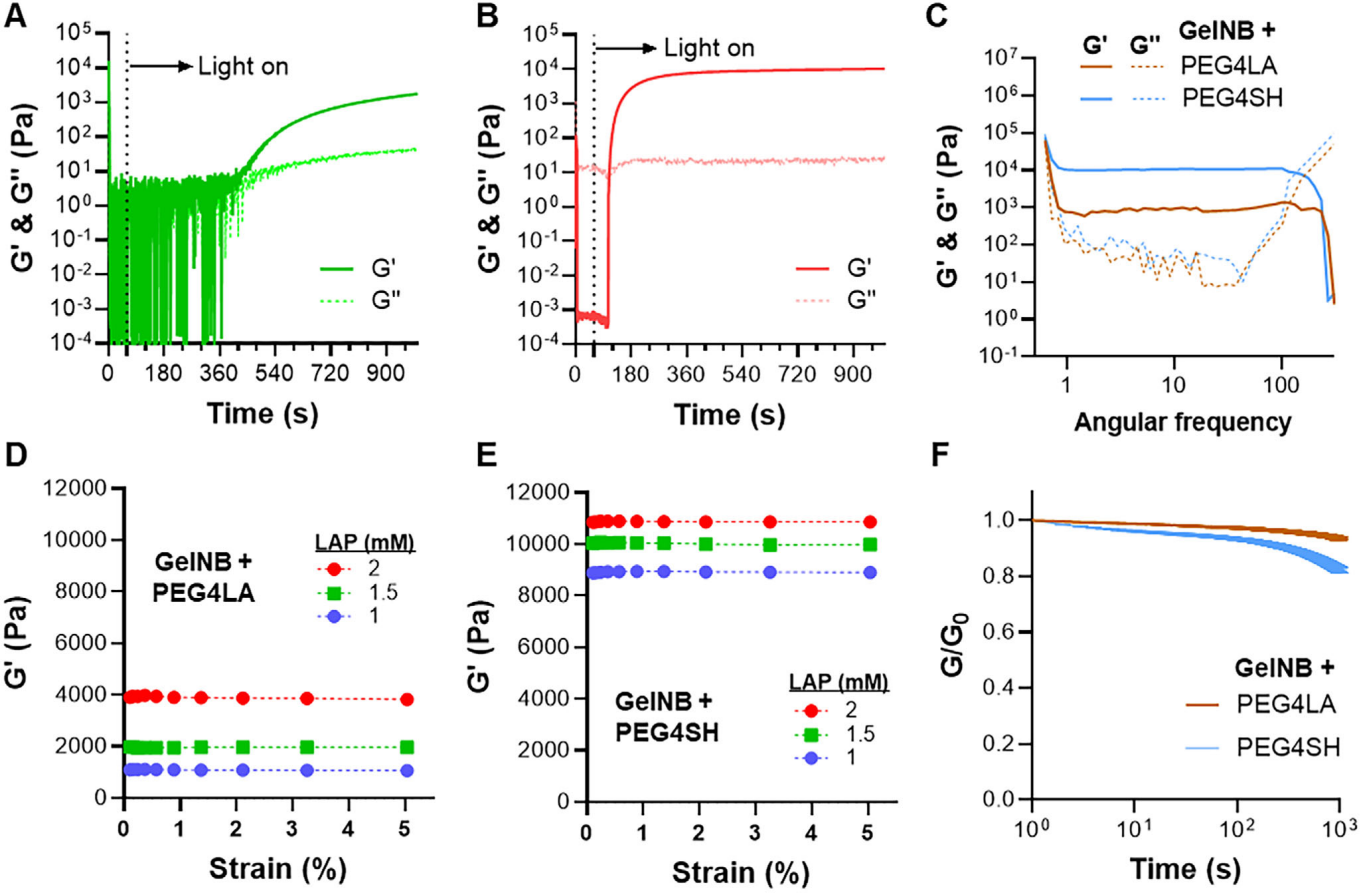
Photopolymerization of dithiolane‐norbornene hydrogels. In situ photo‐rheometry of (A) Photoinitiator‐free dithiolane‐norbornene crosslinking (365 nm, 5 mW/cm^2^), and (B) photoinitiator‐mediated (1 mm LAP) dithiolane‐norbornene crosslinking (365 nm, 5 mW/cm^2^). (C) Frequency sweep of GelNB/PEG4SH and GelNB/PEG4LA hydrogels. Hydrogels (27 mm NB, 20 mm SH) were crosslinked at 365 nm (5 mW/cm^2^) for 2 min. (D, E) Strain sweep of GelNB/PEG4LA and GelNB/PEG4SH hydrogels crosslinked with varied LAP (1, 1.5, 2 mm) content. (F) Stress relaxation behavior of GelNB/PEG4SH and GelNB/PEG4LA hydrogels crosslinked at (365 nm, 5 mW/cm^2^) for 2 min.

Studies have shown that the addition of norbornene‐containing macromer (e.g., PEGNB) accelerated the crosslinking of dithiolane hydrogels due to the addition of thiyl radical to norbornene (Scheme 1C) [26, 27]. Here, we asked whether norbornene from GelNB could also participate and accelerate the crosslinking of dithiolane gels. We found that mixing GelNB and PEG4LA (both at 5% w/v) in the absence of photoinitiator shortened the G′ and G″ crossover time to ∼360 s (Figure 3A). This accelerated gelation (compared with pure PEG4LA in Figure 2) is attributed to the presence of alkene bonds from norbornene that participate in the ROP of dithiolane. The inclusion of LAP at 1 mm accelerated dithiolane‐norbornene crosslinking, reducing the gel point to ∼60 s (Figure 3B). Increasing the LAP concentration to 2 mm further accelerated the gel point to 10 s (Figure S3).

The mechanical properties of GelNB‐crosslinked non‐dynamic thiol‐ene (with PEG4SH) and dynamic dithiolane‐ene (with PEG4LA) hydrogels were compared using frequency sweep rheometry. Both groups of hydrogels demonstrated a wide range of frequency‐independent (1–100 Hz) linear viscoelastic region (LVR). Within the LVR, thiol‐ene and dithiolane‐ene hydrogels exhibited higher storage moduli than loss moduli, suggesting that the gels remained largely elastic solids (Figure 3C). At an equivalent thiol content of 20 mm SH in the precursor solution, GelNB hydrogels crosslinked by PEG4LA were softer than those crosslinked by PEG4SH regardless of LAP concentration (Figure 3D,E). This could be attributed to differences in the molecular weight of the PEG crosslinkers (10 kDa for PEG4SH and 20 kDa for PEG4LA) or incomplete ROP during the short gelation time (2 min) with low light intensity (5 mW/cm^2^). In GelNB/PEG4SH thiol‐norbornene gels, increasing GelNB (5.4 mm of NB/wt%) from 2.5% to 5% led to a substantial increase in storage moduli (G’ ∼1.6–8.4 kPa, Figure S4A). Further increasing GelNB to 10% did not yield stiffer gels (G’ ∼8.4–7.1 kPa, Figure S4A), most likely due to a low thiol‐to‐ene ratio (ca. ∼0.37) that typically resulted in weaker gels. However, the same phenomenon was not observed in GelNB/PEG4LA dithiolante‐norbornene hydrogels, as increasing GelNB to 10% w/v led to stiffer gels (Figure S4B). This result suggests incomplete ROP during dithiolane‐norbornene crosslinking. Additional control experiments with a higher thiol content (40 mm SH or a thiol‐ene ratio of 0.185) in the GelNB/PEG4SH formulation failed to crosslink within 2 min (Figure S4C). In contrast, the same thiol concentration (40 mm) in GelNB/PEG4LA resulted in successful gelation, producing gels with G’ of ∼2,000 Pa (Figure S4D). Finally, stress‐relaxation tests were performed on GelNB/PEG4SH and GelNB/PEG4LA hydrogels, as some dithiolane‐based hydrogels have been shown to exhibit stress‐relaxation behaviors [27, 29]. Interestingly, both hydrogels showed limited stress relaxation within 1,000 s, indicating a more elastic network (Figure 3F). This was to be expected, as Nelson et al. showed that PEGNB/PEGLA hydrogels crosslinked with a 1:2 ratio of NB to LA led to gels with no stress relaxation [27].

### 3.4 Stiffening of Gelatin‐Based Dithiolane‐Norbornene Hydrogels

**FIGURE 4:**
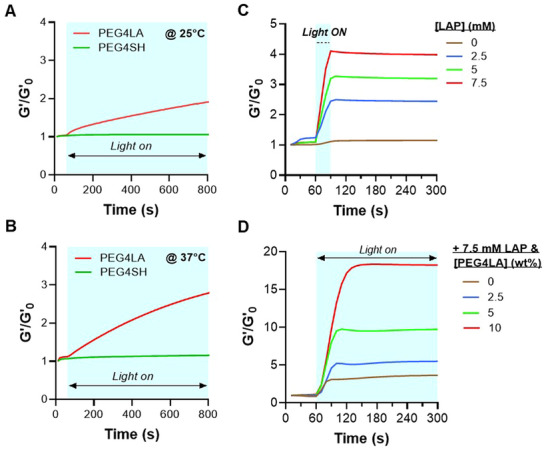
Dynamic stiffening of dithiolane‐norbornene hydrogels. Stiffening of GelNB hydrogels via additional light exposure (365 nm, 5 mW/cm^2^) at (A) 25°C and (B) 37°C. PEG4SH or PEG4LA with equivalent total thiol content was used as the crosslinker. (C) Initiator‐mediated stiffening of GelNB/PEG4LA hydrogels at 25°C. (D) Effects of secondary PEG4LA incubation on stiffening of GelNB/PEG4LA hydrogels. Pre‐crosslinked GelNB/PEG4LA (5% w/v each) hydrogels were incubated in 7.5 mm LAP along with PEG4LA for 60 min at 37°C, followed by secondary light exposure (365 nm, 5 mW/cm^2^).

The disulfide bonds in PEG4LA were exploited to modulate the crosslinking density of GelNB/PEG4LA hydrogels. As disulfide ROP is temperature‐dependent [25], we tested the effect of temperature (25°C and 37°C) on light‐induced stiffening of GelNB hydrogels, either crosslinked by PEG4LA or PEG4SH. Additional light exposure did not increase the stiffness of thiol‐norbornene hydrogels at either temperature (PEG4SH in Figure 4A,B). On the other hand, further light irradiation (without adding any initiator or macromer) resulted in a gradual increase in the stiffness of dithiolane‐norbornene hydrogels (PEG4LA in Figure 4A,B), leading to ∼2 and ∼3‐fold of G’ at 25°C and 37°C, respectively, at the end of 740 s (Figure 4A,B). This was likely caused by additional disulfide ROP post‐gelation [25].

GelNB/PEG4LA dithiolane‐norbornene hydrogels (5% each) crosslinked by 1 mm LAP (G_0_’ ∼ 1 kPa) were further evaluated for dynamic stiffening. Hydrogels were incubated in buffers containing LAP (2.5, 5, 7.5 mm), followed by additional light exposure at 25°C. Upon turning on the light, the moduli of the GelNB/PEG4LA hydrogels increased almost instantaneously (Figures 2 and 3), indicating that stiffening within a preexisting network is easier than forming a new network. These results further support the notion that ROP during primary crosslinking is incomplete and that the network remains capable of undergoing additional dynamic disulfide exchange and thioether formation, which can be accelerated by a photoinitiator. The fold change in moduli was positively correlated with exogenous LAP content, with 2.5‐, 5‐, and 7.5‐mm LAP yielding ∼2.4‐, 3.2‐, and 4‐fold increases in G’, respectively (Figure 4C). The degree of hydrogel stiffening was also positively correlated with exogenous PEG4LA content at a fixed LAP concentration (7.5 mm). Specifically, hydrogels incubated in 2.5, 5, and 10% w/v PEG4LA were stiffened to ∼5.5‐, 9.7‐, and 18.2‐fold relative to their initial moduli (Figure 4D). The substantial increases in G’ (i.e., from ∼1 to ∼18 kPa with 10 w/v% PEG4LA) were notable for the GelNB/PEG4LA hydrogel system. Previously, Perera and Ayres synthesized a linear thiol‐bearing polymer to crosslink GelNB into thiol‐norbornene hydrogels. The abundant thiols on the linear polymer crosslinker enable dynamic stiffening by infusing bifunctional PEGNB and the photoinitiator I‐2959 [34]. However, fine‐tuning of polymer architecture was needed to achieve a modest degree of stiffening (∼34% increase). In contrast, our GelNB/PEG4LA hydrogel system is synthetically simple yet affords a higher degree of stiffening (2‐ to 18‐fold) even in the absence of additional gelation components (Figure 4A,B).

### 3.5 Softening of Gelatin‐Based Dithiolane‐Norbornene Hydrogels

**FIGURE 5:**
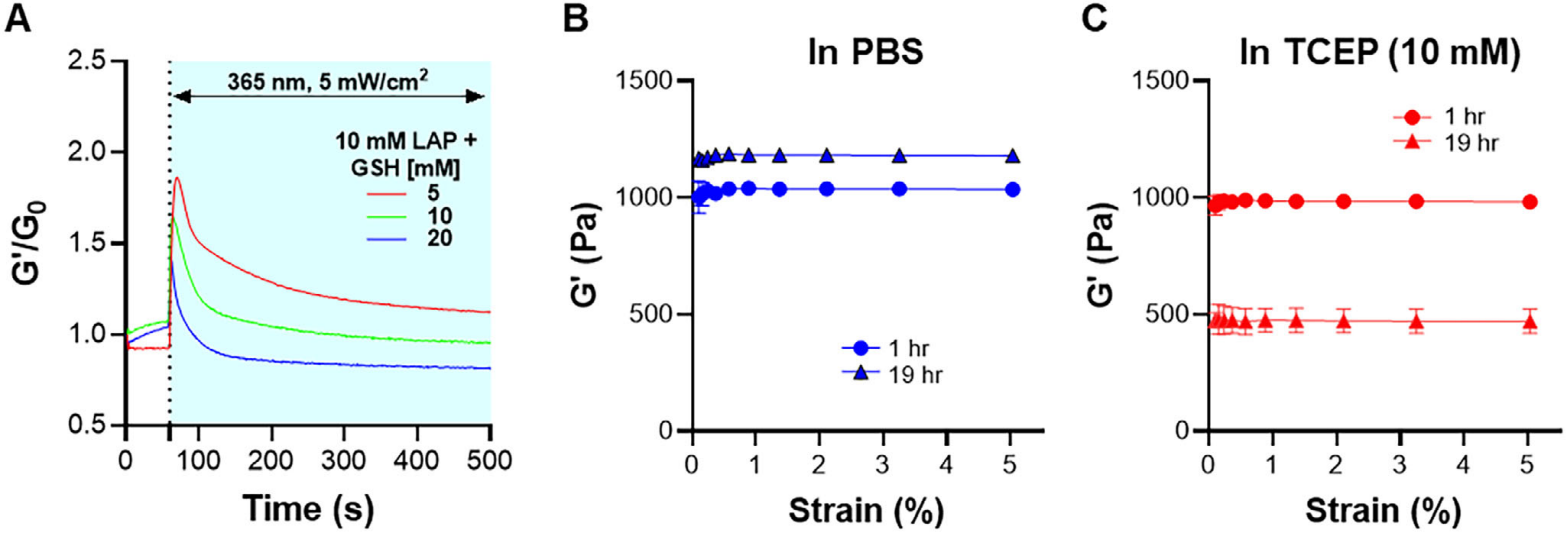
Dynamic softening of dithiolane‐norbornene hydrogels. (A) Light‐based softening of GelNB/PEG4LA (5% w/v each) gels in the presence of LAP (10 mm) and varied Glutathione concentrations (5, 10, 20 mm). (B) Shear moduli of GelNB/PEG4LA hydrogels incubated in PBS for 1 and 19 h. (C) Shear moduli of GelNB/PEG4LA hydrogels incubated in 10 mm TCEP for 1 and 19 h. All hydrogels were crosslinked by 1 mm LAP.

Next, we assessed the softening of GelNB/PEG4LA hydrogels via supplying additional LAP, glutathione (GSH), and light irradiation. GSH was required because its free thiol would terminate reactive thiyl radicals generated by LAP‐mediated disulfide cleavage, thereby decreasing gel crosslinking density and softening [25]. To achieve this, GelNB/PEG4LA hydrogels were incubated in PBS containing either 10 or 30 mm LAP, followed by 365 nm light irradiation at 5 mW/cm^2^. Gel moduli were monitored during light irradiation using time‐sweep rheometry and normalized to the pre‐irradiation value. Interestingly, the gels did not soften immediately but instead stiffened upon illumination, and the increase in G’ was inversely proportional to GSH concentration (Figure 5A; Figure S5A). Following the sharp increase in G’, gradual and GSH concentration‐dependent reductions of moduli were observed (Figure 5A; Figure S5A). Increasing LAP concentration to 30 mm LAP did not alter the sequential stiffening‐softening patterns (Figure S5A). These findings suggest that light‐induced dithiolane gel softening is limited because the GSH‐thiyl radical combination event (responsible for softening) competes with thiyl‐disulfide exchange. While softening may be achieved by using much higher LAP concentrations, this approach is not favorable due to potential cytotoxicity concerns associated with ultra‐high radical concentrations.

Because LAP and GSH did not produce significant gel softening under low light intensity (365 nm, 5 mW/cm^2^), we sought to determine the extent to which these GelNB/PEG4LA gels can be softened. TCEP, a thiol‐free disulfide‐reducing agent, was used in this study. TCEP reduces disulfide bonds not through thiol‐disulfide exchange but by a nucleophilic attack of the phosphorus atom on the disulfide bond. In the presence of sufficient TCEP, all disulfide bonds in the hydrogels would be reduced, causing the reduction of hydrogel crosslinking density. We tested the effect of TCEP on hydrogel stiffness 1 and 19 h post‐TCEP addition, with TCEP‐free PBS as the control. GelNB/PEG4LA hydrogels crosslinked with 1 mm LAP were ∼1 kPa before TCEP incubation. After 19 h of incubation in PBS, the elastic moduli of GelNB/PEG4LA hydrogels increased by ∼150Pa (Figure 5B), likely caused by a small portion of disulfide exchange between dithiolanes across different PEG arms. On the other hand, G’ of gels incubated in 10 mm TCEP decreased by ∼54% (Figure 5C), indicating that roughly half of the original crosslinks were from radical‐mediated disulfide exchange and the other half were thioether bonds. GelNB/PEG4LA hydrogels crosslinked by higher LAP concentration (e.g., 5 and 10 mm LAP) were substantially stiffer (G’ ∼8.5 kPa). However, 19 h of TCEP treatment led to smaller decreases in G’, ∼25% and 30% for gels crosslinked by 5 and 10 mm LAP, respectively (Figure S5B,C). Based on the rubber elasticity theory, gel moduli are proportional to crosslinking density (*ρ*) and take the form of *G* = *ρ_x_k*_B_ *T* [35, 36]. In GelNB/PEG4LA hydrogels, the total *ρ_x_* is contributed by both thioether and disulfide bonds, which can be reduced with TCEP. Hence, the difference in gel moduli before and after TCEP treatment can be attributed to the presence of disulfide bonds. The smaller reductions in G’ for GelNB/PEG4LA hydrogels crosslinked with higher LAP concentrations would indicate that these stiffer gels may contain a smaller proportion of disulfide bonds relative to thioether bonds within the network. This study illustrates that, while disulfide bonds in dithiolane‐norbornene hydrogels can be reduced, the presence of thioether bonds can restrict the reversibility of disulfide‐ene linkages within the hydrogel network.

### 3.6 3T3 Culture in Dynamic Dithiolane‐Norbornene Hydrogels

**FIGURE 6:**
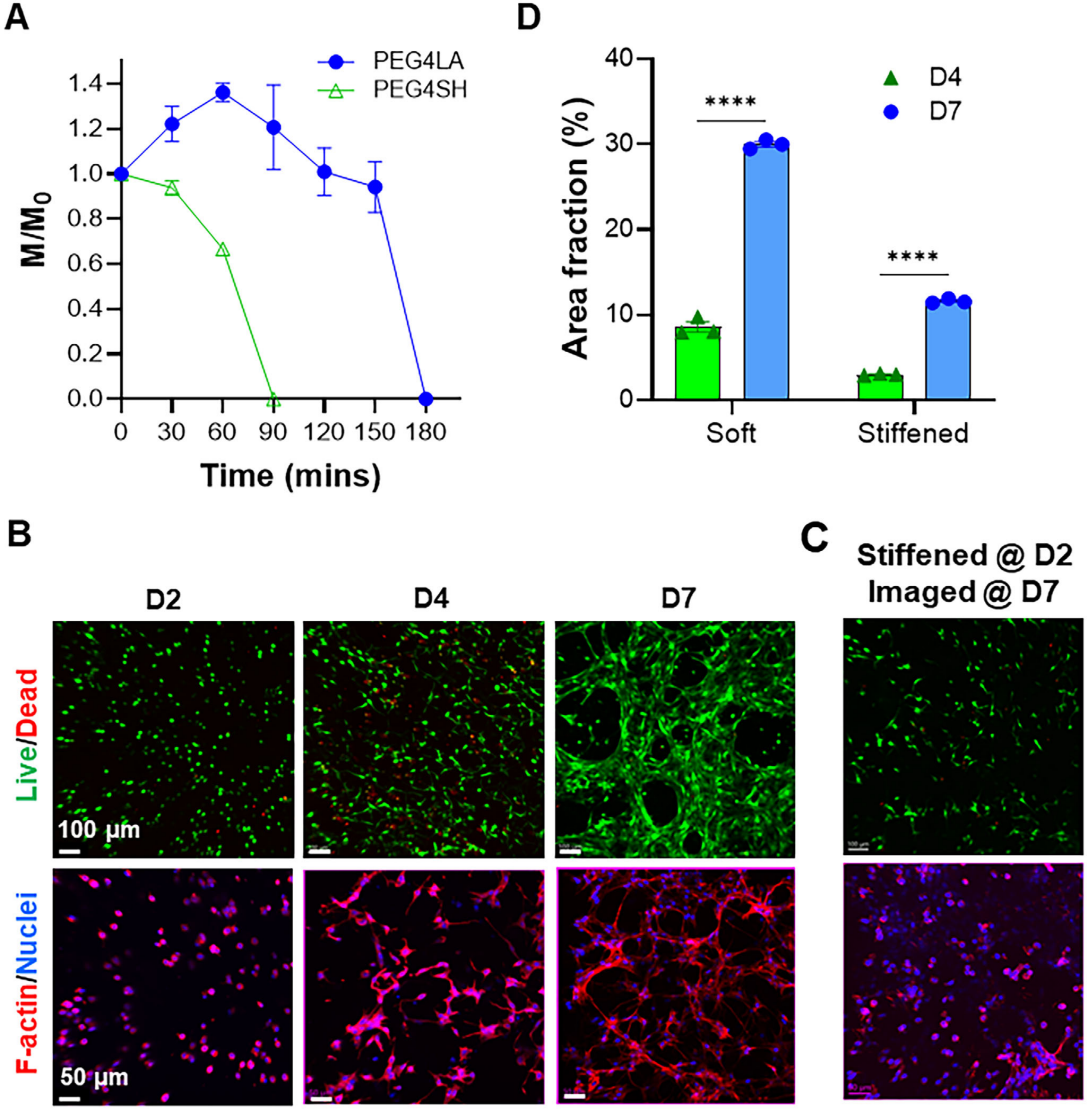
Dynamic stiffening of GelNB/PEG4LA hydrogels on 3T3 cells spreading in 3D. (A) Enzymatic degradation of GelNB/PEG4SH and GelNB/PEG4LA hydrogels using collagenase (100U/mL). (B, C) Live/dead and F‐actin staining of 3T3 cells before stiffening (B) and after dynamic stiffening (C). (D) Spreading of 3T3 cells in soft and stiffened hydrogels over 7 days. F‐actin images acquired via confocal microscopy were analyzed using ImageJ. Four asterisks (^****^) denote *P* ≤ 0.0001.

Dynamically stiffened hydrogels provide a means to investigate how matrix stiffening influences protease‐mediated matrix degradation and subsequent cell spreading. Protease‐mediated hydrogel degradation was characterized by monitoring hydrogel mass changes over time (i.e., M/M_0_, where M_0_ and M represent hydrogel mass before and during enzymatic treatment, respectively). At the onset of collagenase I‐mediated degradation, M/M_0_ equals 1 for both groups of gels. For PEG4SH‐crosslinked thiol‐norbornene hydrogels, the hydrogel mass decreased rapidly after adding collagenase and reached 0 (i.e., M/M_0_ = 0) within 90 min. At the same amount of protease‐labile GelNB (at 5% w/v), PEG4LA‐crosslinked dithiolane‐norbornene hydrogels degraded more slowly than the thiol‐norbornene counterparts (Figure 6A). Initial enzyme treatment increased M/M_0_ to above 1, suggesting that more water infiltrated the hydrogels. The entire hydrogel eventually degraded (i.e., M/M_0_ = 0) after sufficient chemical bonds had been degraded. At 5% GelNB, the concentration of protease‐labile motifs should be the same for both PEG4SH and PEG4LA crosslinked gels. The vastly different degradation behaviors suggest that these two types of gels might have different network connectivity (i.e., 4 functional groups per PEG4SH and 8 functional groups per PEG4LA).

To determine whether delayed protease‐mediated degradation would hinder cell spreading in these hydrogels, we encapsulated mouse embryonic fibroblasts (3T3 cells) in GelNB/PEG4LA hydrogels. Live/dead staining results showed excellent cytocompatibility of GelNB/PEG4LA hydrogels in 3T3 cultures in both the control and stiffened groups (Figure 6B). Before stiffening, cells exhibited rounded morphology (Figure 6B). Over 7 days of culture, soft GelNB/PEG4LA hydrogels supported the formation of interconnected spindle‐like morphology of 3T3 cells, whereas cell spreading was limited in the stiffened hydrogels (Figure 6C). Quantitative analysis of viability images demonstrated statistically significant differences in 3T3 spreading in soft and stiffened gels (Figure 6D). 3T3 cells in soft gels exhibited progressive spreading, reaching about 35% area fraction (proportion of the image occupied by cells relative to the total image area) after 7 days (Figure 6C,D; Figure S6). In contrast, cells in stiffened gels exhibited slower spreading, reaching about 10% area fraction after 7 days (5 days post‐stiffening) (Figure 6C,D). This observation is consistent with previous studies where soft hydrogels promoted greater cell spreading compared to stiff or dynamically stiffened gels [25].

### 3.7 iPSC Culture and Differentiation in Dynamic Dithiolane‐Norbornene Hydrogels

**FIGURE 7:**
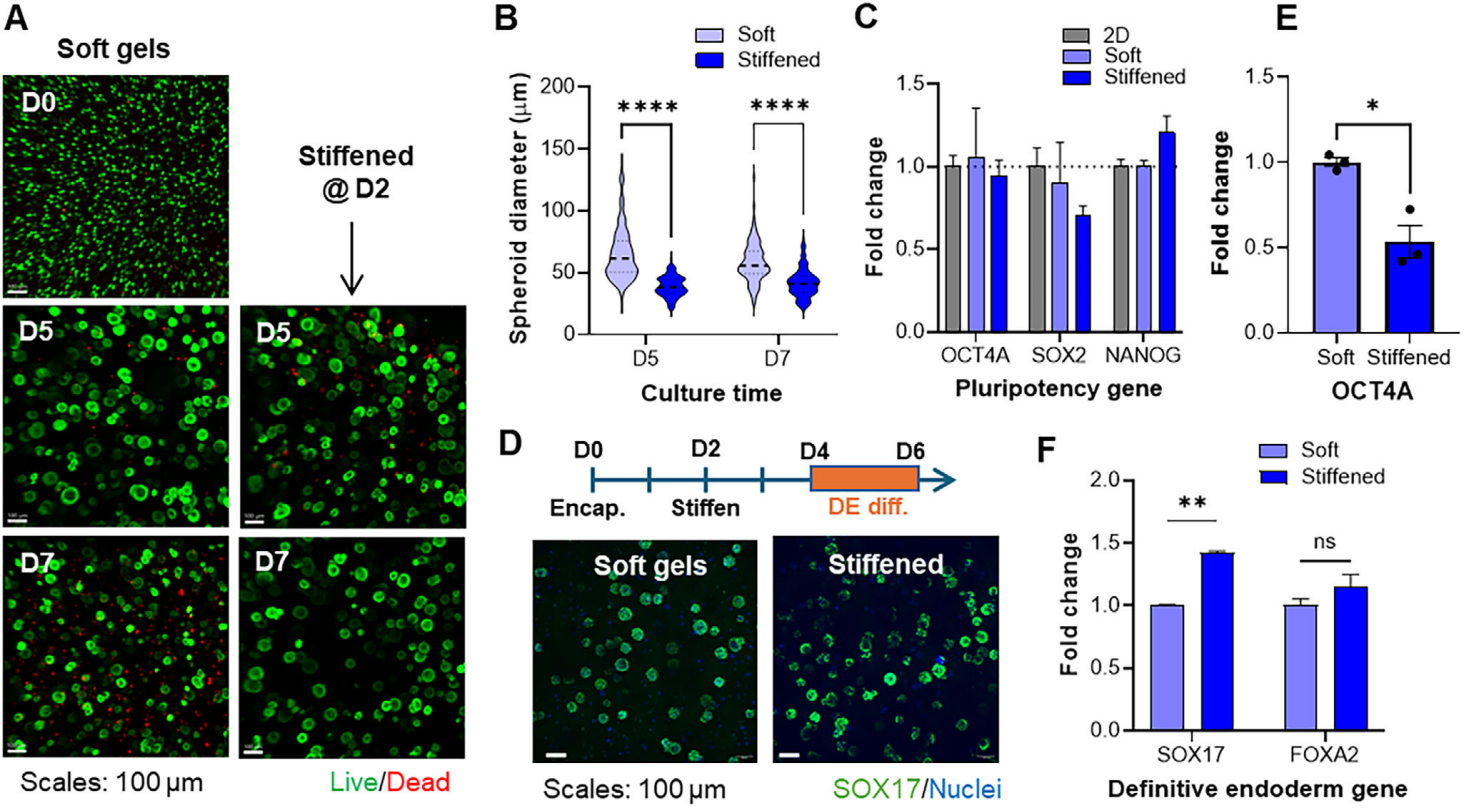
Dynamic stiffening of GelNB/PEG4LA hydrogels on iPSC viability and pluripotency. (A) Live/Dead staining and images of ChiPSC12 cells in static soft and dynamically stiffened hydrogels. Dynamic stiffening was induced on day 2 of culture via gel incubation in 2.5 mm LAP added to the media, followed by photoirradiation. (B) Quantification of iPSC spheroid diameters without or with dynamic stiffening. (C) Expression of pluripotency makers in ChiPSC12 cells encapsulated in GelNB/PEG4LA hydrogels. Cells cultured on 2D plastics were used as a control. All gene expressions were normalized internally to GAPDH. (D) Timeline of dynamic gel stiffening and DE differentiation, as well as immunostaining of a DE marker, SOX17, in ChiPSC12 cells encapsulated in soft and dynamically stiffened GelNB/PEG4LA hydrogels. (E) mRNA expression of pluripotency gene OCT4A after DE differentiation in soft and dynamically stiffened hydrogels. (F) mRNA expression of DE genes SOX17 and FOXA2 after two days of DE differentiation. One asterisk (^*^) denotes *P* ≤ 0.05, two asterisks (^**^) denote *P* ≤ 0.01, four asterisks (^****^) denote *P* ≤ 0.0001.

Studies have shown that hydrogels with higher stiffness (E ∼ 6–7 kPa or G’ ∼ 2.5 kPa) promoted endodermal differentiation [37, 38]. To this end, ChiPSC12 cells in GelNB/PEG4LA hydrogels (G’ ∼ 1 kPa) were highly viable following encapsulation (D0) and formed larger aggregates over 7 days of culture (Soft gel, Figure 7A). Dynamic gel stiffening with additional LAP (2.5 mm) and light exposure did not reduce cell viability (Stiffened @ D2, Figure 7A), but decreased the size of cell spheroids (Figure 7A,B). Specifically, the diameters of spheroids in soft gels and dynamically stiffened hydrogels were ∼40 and ∼62 µm, respectively (Figure S7A). Notably, ChiPSC12 spheroids in dynamically stiffened hydrogels exhibited a narrower size distribution than those in soft gels, as reflected by a lower standard deviation in the dataset (Figure 7B; Figure S7A). In addition, spheroids formed in GelNB/PEG4LA gels were smaller overall than previously reported in a PEG‐based platform [38], potentially due to the longer time needed for the proteolytic degradation of GelNB/PEG4LA gels (Figure 6A).

Regardless of the hydrogel stiffening condition, hiPSCs maintained pluripotency comparable to that in 2D cultures, as confirmed by qPCR analysis of pluripotency markers OCT4A, SOX2, and NANOG (Figure 7C; Table S1). Next, we performed DE differentiation using a commercially available kit (STEMCELL Technologies). Following the same encapsulation timeline as undifferentiated hiPSCs, we initiated DE differentiation on day 4 of encapsulation (i.e., 2 days after stiffening) and continued for 2 days (Figure 7D). Cells remained viable in both soft and stiffened gels at the end of differentiation, with minimal differences observed (Figure S7B). However, some cells lost the rounded morphology observed in their undifferentiated counterparts, indicating early morphological changes associated with lineage commitment/differentiation (Figure S7B). Immunostaining revealed notable expression of SOX17, confirming successful DE differentiation within the hydrogels (Figure 7D). qPCR analysis further showed downregulation of the pluripotency marker OCT4A in stiffened gels (Figure 7E) and significantly higher expression of the DE marker SOX17 in dynamically stiffened gels compared with soft gels (Figure 7F).

Prior studies have demonstrated that stem cell viability and pluripotency can be maintained in static hydrogel environments of optimized stiffness and degradability. For instance, Ovadia et al. achieved moderate iPSC viability in PEGNB hydrogels [39], while Hubbell [40] and Lim [41] showed that PEG‐vinyl sulfone (PEG‐VS) hydrogels supported long‐term pluripotency and proliferation of mouse and human ESCs through integrin‐mediated signaling and matrix remodeling. Similarly, our group has demonstrated that GelNB thiol‐norbornene hydrogels supported hiPSC proliferation, pluripotency [18], and robust differentiation into neuroectoderm, mesoderm, and definitive endoderm [18]. Although these studies highlight the importance of matrix mechanics in supporting stem cell fate, they are limited by static mechanical environments. Increasing evidence indicates that stem cell niches are inherently dynamic, with continuously evolving biochemical and biophysical cues that regulate cell behavior and tissue morphogenesis during development [42]. Hence, dynamic hydrogels with high cytocompatibility for in situ encapsulation of hiPSCs are highly desirable [42]. This work describes a gelatin‐based dynamic hydrogel system by leveraging dithiolane‐norbornene photochemistry, a strategy previously explored only in inert PEG‐based hydrogel systems [27, 30]. Unlike conventional static thiol‐ene or enzymatically crosslinked gelatin systems, the dithiolane‐norbornene reaction enables reversible network rearrangement while maintaining cytocompatibility, allowing us to decouple hydrogel stiffness and early lineage specification. To our knowledge, no prior dithiolane‐norbornene hydrogels have been investigated for dynamic differentiation of hiPSCs. The in vitro cell studies presented in this work demonstrate the high cytocompatibility of GelNB/PEG4LA hydrogels for in situ differentiation of hiPSCs.

## 4 Conclusion

Conventional gelatin‐based hydrogels lack dynamically tunable properties, unless multiple modifications are made to gelatin. This study addresses this issue by combining GelNB and PEG4LA, both of which were synthetically simple but, when crosslinked together, formed a cell‐responsive and highly dynamic matrix. However, unlike conventional GelNB/PEG4SH thiol‐norbornene hydrogels, GelNB/PEG4LA dithiolane‐norbornene gels were less susceptible to proteolysis, likely due to the steric hindrance of multiple disulfides formed in the gels. Unlike pure dithiolane hydrogels, the disulfide linkages in GelNB/PEG4LA could not be readily de‐crosslinked, and further studies are needed to elucidate the underlying mechanisms. Nonetheless, we demonstrated the cytocompatibility of GelNB/PEG4LA hydrogels for in situ culture and DE differentiation of hiPSCs. Future work will leverage the dynamic stiffening capacity of GelNB/PEG4LA hydrogels to explore mechano‐regulation in stem cell fate commitment and to expand this system to engineer biofabricated constructs that support and enhance the differentiation of iPSC‐derived cells.

## Conflicts of Interest

The authors declare no conflict of interest.

## Supporting information




**Supporting File**: mabi70149‐sup‐0001‐SuppMat.docx.

## Data Availability

The data that support the findings of this study are available in the supplementary material of this article.
