# Supplementary material for: Dynamic Gelatin Hydrogels Crosslinked by Dithiolane‐Norbornene Click Chemistry
Source: Macromol Biosci. 2026 Feb 3;26(2):e00400. doi: 10.1002/mabi.202500400 (PMC12868403; doi:10.1002/mabi.202500400)
Supplement: Supplementary file 1 — Supporting File: mabi70149‐sup‐0001‐SuppMat.docx. [file MABI-26-e00400-s001.docx]

**Dynamic gelatin hydrogels crosslinked by dithiolane-norbornene click chemistry**

Favour O. Afolabi, Lydia Yang He, Chien-Chi Lin.

Weldon School of Biomedical Engineering, Purdue University, West Lafayette, IN 47907, USA.

***Supporting information***

*To whom correspondence should be sent:

Chien-Chi Lin, Ph.D.

Professor

Weldon School of Biomedical Engineering

Purdue University

Contact Information:

723 W. Michigan St. SL220K

Indianapolis, IN 46202, USA

Phone: (765) 495-7791

Email: [lin711@purdue.edu](mailto:lin711@purdue.edu)

ORCID: 0000-0002-4175-8796


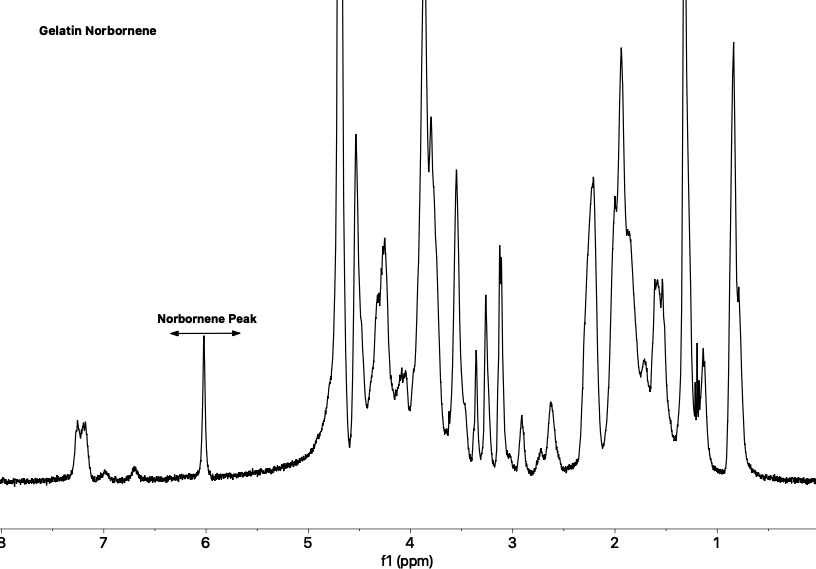


Figure S1. A) ^1^H-NMR spectra of GelNB in deuterium oxide (D_2_O). δ/ppm: 6.11 – 5.93 (protons from norbornene).


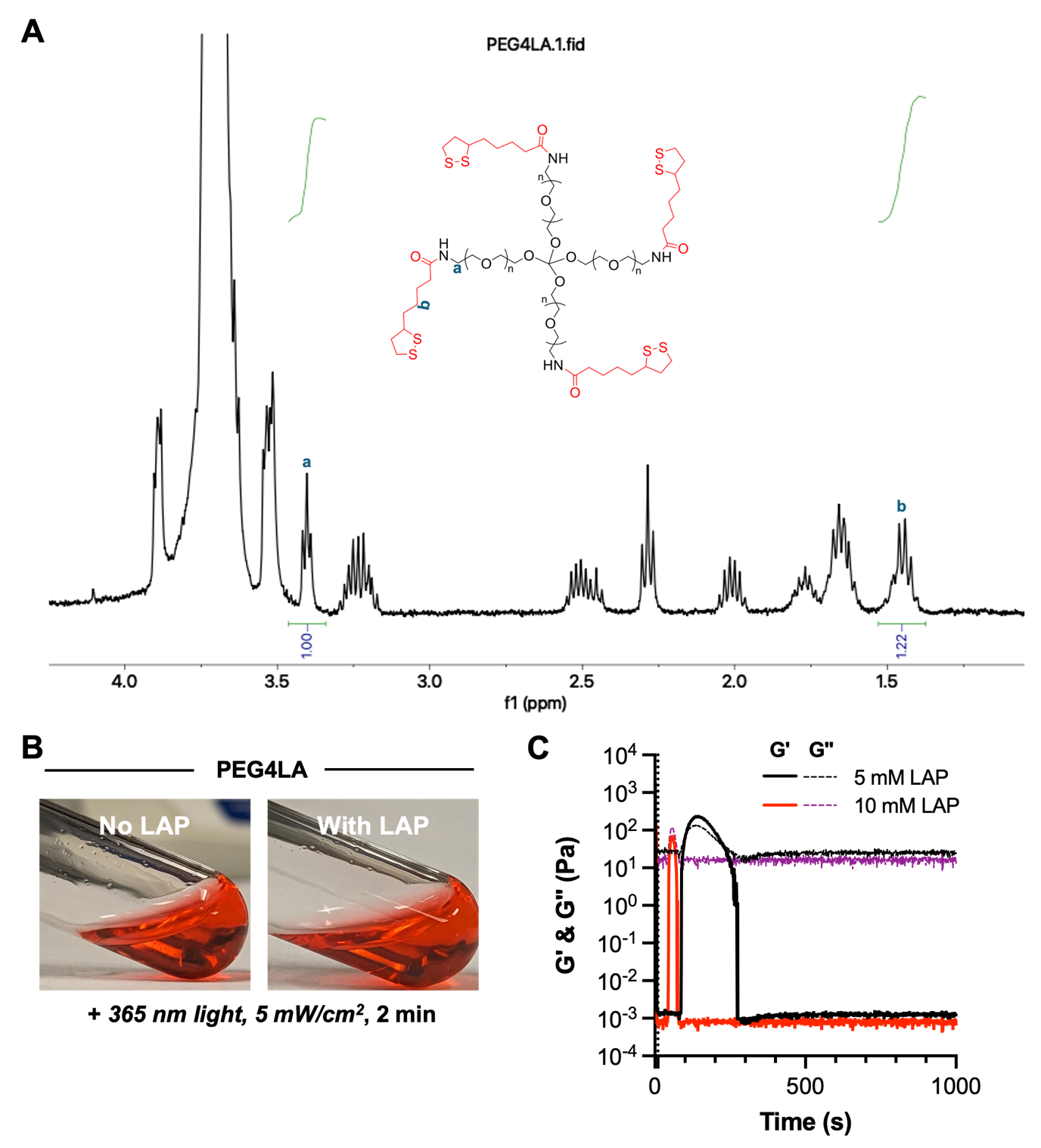


Figure S2. Photo-crosslinking and decrosslinking of PEG4LA. (A) ^1^H-NMR spectra of PEG4LA in deuterium oxide (D_2_O). δ/ppm: 3.93 – 3.36 (protons from PEG-amine), 3.33 – 1.38 (protons from lipoic acid groups). Degree of substitution was determined by comparing the methylene peak from PEG (3.46 – 3.34 ppm) to a methylene peak of lipoic acid (1.53 – 1.37). (B) Vial tilt test of PEG4LA (5 wt%) hydrogel precursor solution crosslinked in the absence and prescence of LAP (1 mM). Red food dye was added to the hydrogel precursor solution for visualization. (C) Initiator-mediated crosslinking and decrosslinking of PEG4LA (5 wt%) crosslinked with LAP ( 5 and 10 mM) at 365 nm (5 mW/cm^2^).


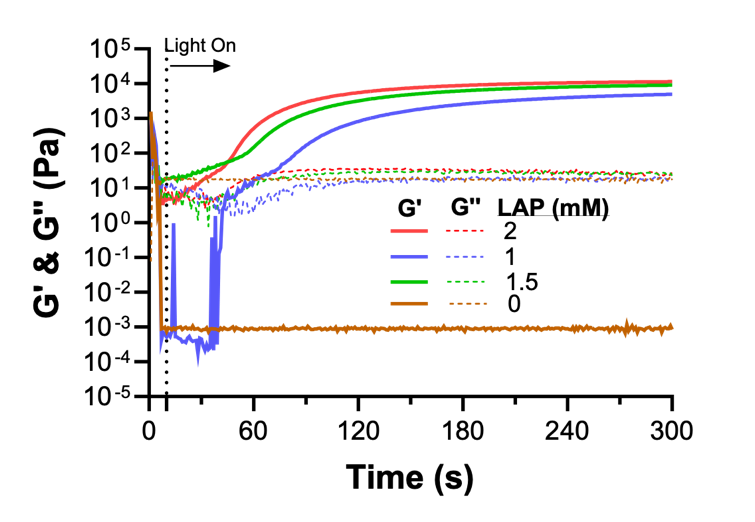


Figure S3. Photo-crosslinking of gelatin-based dithiolane-norbornene hydrogels. Initiator-mediated crosslinking of GelNB/PEG4LA (each at 5 wt%) at low LAP concentrations (1, 1.5, 2 mM) using 365 nm light (5 mW/cm^2^).


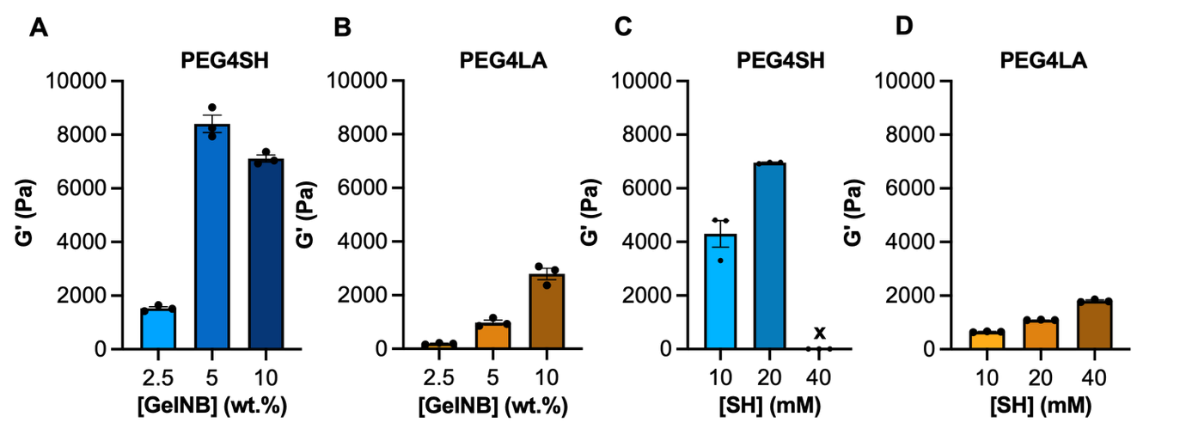


Figure S4. Effects of macromer content on thiol-ene and dithiolane-norbornene hydrogels. (A) Elastic shear moduli of GelNB/PEG4SH and GelNB/PEG4LA hydrogels crosslinked using 365 nm light (5 mW/cm^2^), with varying (A, B) GelNB concentrations (2.5, 5, 10 wt%) and thiol content (10, 20, 40 mM).

**
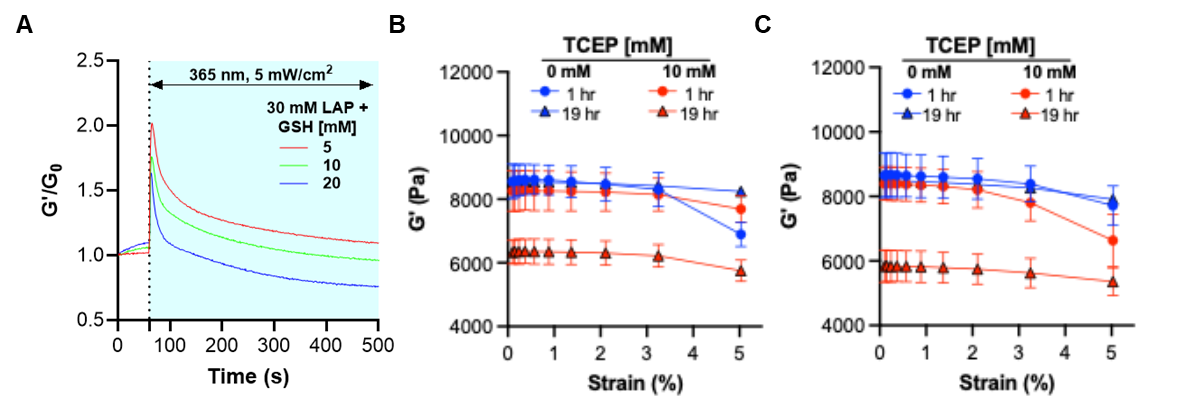
**

**Figure S5. Dynamic softening of dithiolane-norbornene hydrogels.** (A) Light-based softening of GelNB/PEG4LA gels in the presence of LAP (30 mM) and varied Glutathione concentrations (5, 10, 20 mM). (B, C) TCEP (10 mM)-mediated reduction of disulfide bonds in GelNB/PEG4LA (both at 5 wt%) hydrogels crosslinked with 5 mM (B) or 10 mM (C) LAP.


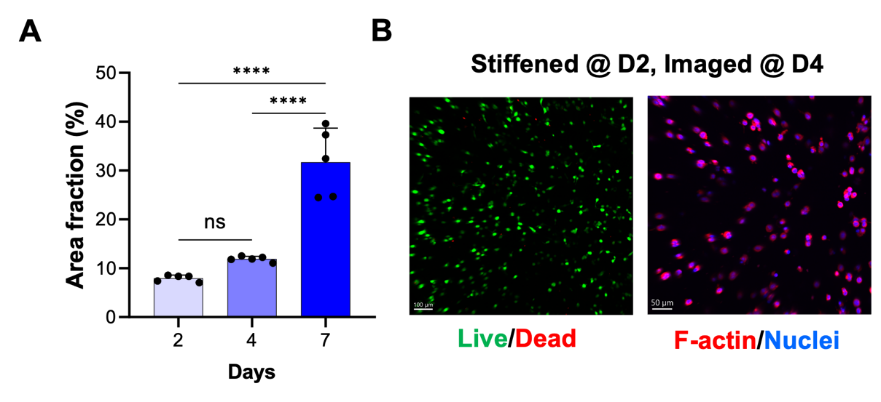


**Figure S6. Impact of dynamic stiffening on hydrogel-laden 3T3 cells.** (A) Spreading of 3T3 cells in soft hydrogels over 7 days. F-actin staining images acquired via confocal microscopy were analysed using ImageJ. (B) Day 4 live/dead and F-actin staining images of 3T3-laden GelNB/PEG4LA hydrogels stiffened at day 2 of encapsulation.


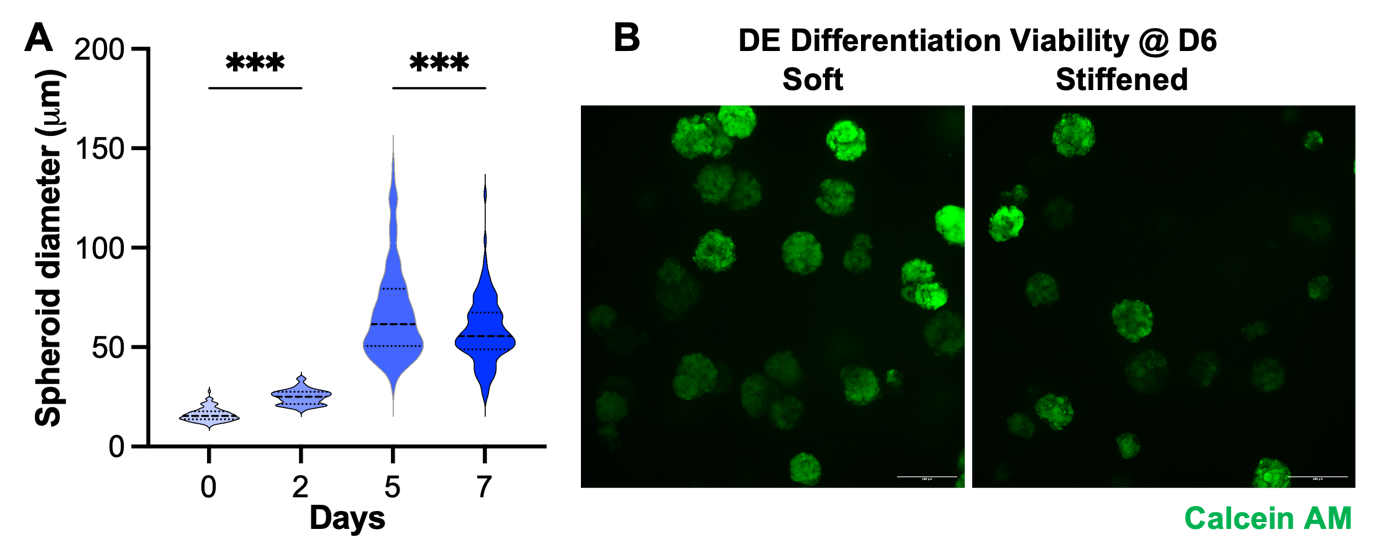


**Figure S7. iPSC growth in GelNB/PEG4LA hydrogels.** (A) iPSC spheroid diameter over seven days of encapsulation. Quantitative analysis was performed on live/dead images collected via confocal microscopy, using ImageJ. Three asterisks (***) denote p < 0.001. (B) CalceinAM staining of iPSCs differentiated to DE cells in soft and dynamically stiffened GelNB/PEG4LA gels.

**Table S1: List of primers used in study.** mGAPDH: housekeeping gene. OCT4A, NANOG, SOX2: pluripotency genes. SOX17, FOXA2: definitive endoderm genes.

| **Gene names** | **Forward primer** | **Reverse primer** |
| --- | --- | --- |
| mGAPDH | ATCACTGCCACCCAGAAGACT | CATGCCAGTGAGCTTCCCGTT |
| NANOG | AAGAACTCTCCAACATCCTGAAC | CCTTCTGCGTCACACCATT |
| SOX2 | GCCGAGTGGAAACTTTTGTCG | GCAGCGTGTACTTATCCTTCTT |
| OCT4A | ATTCAGCCAAACGACCATCT | TCTCACTCGGTTCTCGATACTG |
| SOX17 | GTGGACCGCACGGAATTTG | GGAGATTCACACCGGAGTCA |
| FOXA2 | GGAGCAGCTACTATGCAGAGC | CGTGTTCATGCCGTTCATCC |
